# Maximum strength and power as determinants of on-ice sprint performance in elite U16 to adult ice hockey players

**DOI:** 10.5114/biolsport.2024.129470

**Published:** 2023-08-08

**Authors:** Martin Kierot, Mattia Stendahl, Konstantin Warneke, Klaus Wirth, Andreas Konrad, Torsten Brauner, Michael Keiner

**Affiliations:** 1 Department of Exercise Science, German University of Health & Sport, Ismaning, Germany; 2 Department of Strength and Conditioning, ZSC/GCK Lions Ice-Hockey, Zürich, Swiss; 3 Institute for Exercise, Sport and Health, Leuphana University, Lüneburg, Germany; 4 Faculty of Training and Sports Science, University of Applied Science Wiener Neustadt, Vienna, Austria; 5 Institute of Human Movement Science, Sport and Health, University of Graz, Graz, Austria; 6 Department of Biomechanics, German University of Health & Sport, Ismaning, Germany

**Keywords:** Exercise test, Muscle strength, Athletic performance, Sports, Hockey

## Abstract

In ice hockey, speed strength is one of the major physical key performance indicators, which is significantly influenced by maximum strength. The objective of this study was to evaluate the age-dependent relationship of off-ice maximum strength and vertical jump performance with on-ice linear sprint performance, considering age and performance level. Ninety-one male youth and adult professional ice hockey players (age: 19.3 ± 5.49 years) were recruited and divided into four age groups: under 16, 18, 21 years old and professional elite players (Pro) (i.e., > 21 years). They were tested in maximal isometric strength, squat jump (loaded and unloaded), countermovement jump and on-ice sprint performance (15 m and 30 m linear sprint; 15 m flying linear sprint). Statistical analysis revealed that on-ice sprint performance correlated with isometric strength performance (r = |0.34|-|0.63|) and with off-ice jump performance (r = |0.61|-|0.77|) without an influence of age group or performance level. However, performance differed between age groups and performance level, the largest differences being found between the youngest age group (U16) and the Pro group (g = 0.966–3.281). The present study shows that maximum strength influences on-ice sprint performances in ice hockey players, as well as performance differences between age groups and professional players. Strength and jumping performance should therefore be included in regular performance testing in ice hockey. Since performance differences are observed for almost all strength and speed-strength performances of the youth teams to the Pros, training of these variables is strongly recommended to improve in the transition phase from junior to elite level.

## INTRODUCTION

Maximum and repetitive sprint performance seem to be of high importance in ice hockey [1]. During each minute on ice, a player skates approximately 119 m at high intensity and 31 m at maximum intensity [2]. The total distance covered during a game is about 4600 m, of which 2042 m is at high intensity in roughly 15 min [2]. Therefore, higher sprint performance in 10–30 m sprint seems to be correlated with higher playing level [3, 4], leading to benefits in winning the puck [2, 5].

Therefore, speed strength is a performance determinant which seems to be strongly influenced by maximum strength [6, 7]. Literature investigating the influence of speed strength on on-ice performance is scarce; however, off-ice, studies including elite and recreational team sports athletes other than ice hockey show strong correlations (r = 0.67–0.78) between maximum strength (1 repetition maximum back squat [1RM]) and vertical jumps (countermovement [CMJ] and squat jump [SJ]) as well as linear sprint performance independent of age groups [8–10]. Furthermore, the isometric midthigh pull is an often-used strength test in (sport-) performance tests, showing moderate to high correlations with jumping (r = |0.51–0.73|) [11–14] and sprinting performance (r = |0.53–0.69|) [15, 16]. Studies including male and female college hockey players showed contrasting results ranging from r = |0.21| to |0.74| examining the relationship between different maximum strength performances and on-ice linear sprint (6–44.8 m)) [17–19]. This heterogeneity in correlations could be due to large differences in study design and partially small sample sizes (high sampling error). Additionally, some studies show correlations of r = |0.09|-|0.85| for off-ice performances (different jumps [loaded and unloaded/vertical and horizontal]) with on-ice linear sprint (6–55 m) performances in different performance levels (youth, sub-elite and professional) [5, 17–21].

Similar to other team sports [22–24], in ice hockey [3, 4, 25–27], speed strength is considered to be an important factor differing between age groups or between elite and sub-elite. Players of a Polish professional team were divided into two groups according to their playing level. The group with the best players showed significantly better performances in the 30 m off-ice sprint and in the 6 × 9 m change of direction test compared to the weaker group [25]. Therefore, the authors suggested that speed parameters can be used for the selection of top hockey players [25]. Accordingly, Vigh-Larsen et al. [3] observed significantly better CMJ performance, change-of-direction ability, and linear sprint performance over 0–33 m in professional ice hockey players from the 1^st^ Danish league compared to 2^nd^ league players. Also Bracko & George [26] reported significantly higher performance results in professional compared with amateur female ice hockey players (15 m “on-ice” sprint). The fitness profiles of 204 professional female ice hockey players from 13 countries were compared [28] considering their age (junior < 18 years old or senior group > 18). Compared to the junior group, the senior players performed significantly better in vertical jump, 4-jump, and standing long jump [28]. Hoff et al. [27] analysed the performance of elite (age: 24.2 ± 4.7 years) and junior elite players (age: 17.6 ± 0.9 years), finding significantly better performances in the 1RM back squat, bench press, 10 m sprint, CMJ and SJ with 50 kg in older players.

Although there is already some evidence on the relationship between maximal strength performance and off-ice speed strength performance as well as differences between age and performance groups, there is a need for a further study to get deeper insights for the determining variables for on-ice performance. Therefore, this study was conducted to investigate the influence of maximum strength (absolute and relative maximal isometric strength) using the isometric trap bar pull (ITBP) on vertical jump performance, CMJ, CMJ with an extra 40% of bodyweight (BW), SJ height and “on-ice” linear sprint performance (15 m, 30 m and flying 15 m) in youth elite and professional ice hockey players of the two highest Swiss leagues. Furthermore, differences in performance across age groups, ranging from under 16-year-olds to professional players, were analysed.

## MATERIALS AND METHODS

**FIG. 1:**
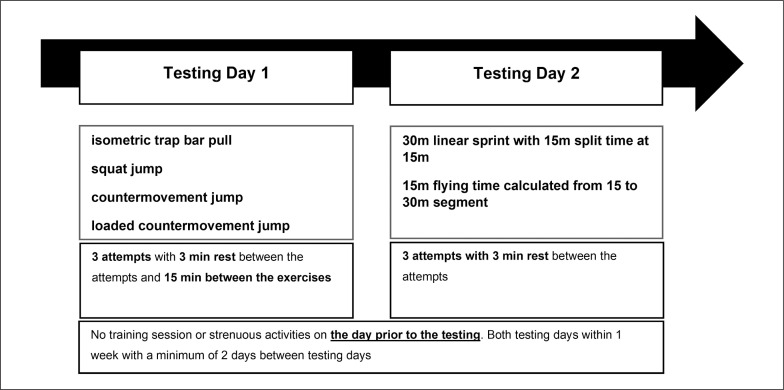
Test procedure.

In order to answer the research question, a cross-sectional study was conducted. To investigate the relationship between performance “off-ice” and performance “on-ice” 91 highly trained male youth and professional ice hockey players were tested off ice in ITBP CMJ, SJ, CMJ 40% BW and maximum speed on-ice (15 m, 30 m, flying 15 m). The tests were performed after two days of rest in the second week of the pre-season. Tests were carried out on 2 test days within a 1-week period. On test day 1 the off-ice tests were performed, followed by the on-ice tests on test day 2 (see Figure 1). A familiarization period was not necessary because all tests are performed at regular intervals (jumps = weekly; strength = monthly, sprint = semiannually) and were therefore known to the players.

### Subjects

Ninety-one male youth and adult ice hockey players (height: 178 ± 6 cm; weight: 75.9 ± 10.8 kg; age: 19.3 ± 5.5 years) competing at the national and international level from a professional ice hockey club in Switzerland were recruited and divided into four (age) groups: U16 (n: 20; height: 175.8 ± 8.1 cm; weight: 66.9 ± 9.4 kg; age: 14.8 ± 0.4 years), U18 (n: 28; height: 177.9 ± 5.9 cm; weight: 71.7 ± 7.9 kg; age: 16.4 ± 0.5 years); U21 (n: 22; height: 178.6 ± 5.2 cm; weight: 80.2 ± 9.2 kg; age: 18.8 ± 1.0 years); Pro (n: 21; height: 180.1 ± 5.3 cm; weight: 85.7 ± 6.8 kg; age: 28.4 ± 3.9 years). The players of the Pro subgroup play in the highest and the U21 in the second highest national league. Five players of the subgroup Pro are A national team players. For the U18 and U16, players were recruited who play in the highest junior leagues in their respective age groups. Of these, 14 players also play for the junior national teams. Training volume is stated with 14 hours per week (h/w) during the off season in both professional teams (Pro and U21) and 10 hours per week (h/w) in all youth teams. In the preseason and in-season, the training volume varies between 6 and 10 hours/week and an additional 1 to 3 games per week in all subjects. The subjects did not participate in fatiguing training sessions for a minimum of 2 days prior to testing. None of the subjects reported any injuries at the time of testing. Each subject and their parents (for underaged participants) were informed about the aims of the study and the experimental risks involved with the research and provided written informed consent. Furthermore, this study was performed in accordance with the Helsinki Declaration and was approved by the Universities Ethics Committee (DHGS-EK-2021-002).

### Procedure and Measurements

#### Off-ice Tests

**FIG. 2:**
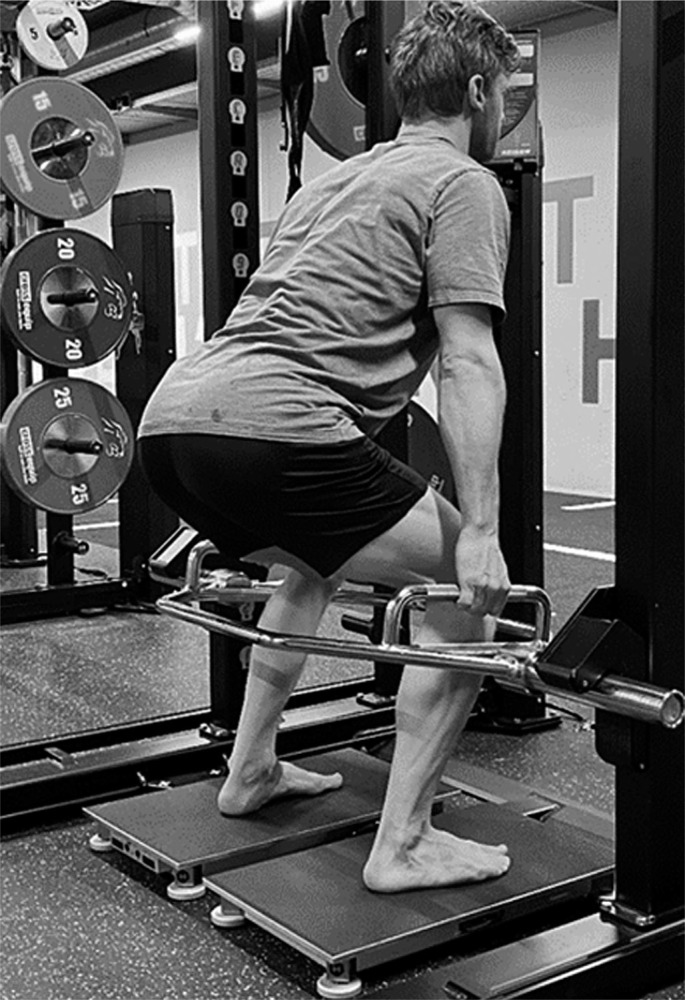
Isometric trap bar pull.

All strength and power tests were performed on a split force plate (Hawkin Dynamic Wireless Dual Force Platform V.3.0., Main, USA). All participants completed a standardized 20 min warm-up protocol before the off-ice tests. The protocol included glute bridges, various mini band exercises to activate the hip muscles, 10 repetitions each of BW split squats, BW squats, SJ, and CMJ. After the standardized warm-up, the maximum strength was first determined with the ITBP (Figures 1 and 2). Here, 3 trials were performed with a rest period of 3 min between attempts. After another breaks of 15 min, the SJ and CMJ were tested, each with three attempts. Maximum concentric power can be generated using a range of external loads between 30% and 60% of maximum strength [21, 29]. Therefore, also 3 attempts for CMJ 40% BW were performed after another break of 15 min.

The ITBP was performed with a hip-width start position and with minimal outward rotation of the feet allowed. The athlete held the trap bar under fixed pins attached to the rack. In this position, the knee angle was determined by a mobile app (Coach’s Eye, Tech-Smith Corporation) and protractor. If a knee angle of 90 degrees was measured, the athlete was allowed to start the first attempt after a brief pause. Once the test started, the athlete was instructed to increase the pull to a maximum against the pins over 5 seconds (s). If the athlete could not keep their back straight, the attempt was considered invalid. The highest value in N and N per kg of BW (N/kg) was used for the data evaluation. This protocol was very similar to the one used by another study in the field of ice hockey, where maximum isometric strength was assessed [30]. The intersession reliability is assumed with ICC = 0.99 [30].

The jump height was calculated with take-off velocity^2^ / (9.81*2) and from flight time (gt^2^/8; with g being the gravitational acceleration (9.81 m·s^−2^) and t = flight time in seconds) via software (Hawkin Dynamic, Main, USA). For the SJ the athlete placed the left foot on the left plate and the right foot on the right plate. The jump was only scored if the body travelled directly upwards. If a downward movement to the ground was executed before take-off the jump was scored as invalid. Likewise, a jump was considered invalid if the athlete removed their hands from their hips during the jump. The same criteria applied to the CMJ; however, a countermovement should be induced. Interclass reliability for similar test setups is assumed with ICC = 0.87–0.98 [31, 32]. In the CMJ with extra 40% BW, the athlete with the loaded trap bar also placed themself on the plate. In general, the exact same conditions were chosen as for the classic CMJ. Additionally, the trap bar was gripped centrally, with a neutral grip, and a neutral spine position was assumed. The weight could be adjusted to within 500 g of the calculated value. Interclass reliability following other studies with similar protocol is assumed with an ICC = 0.92 [33].

#### On-ice Tests

On the ice 5 min (submaximal skating) warm up was performed including two 30 m acceleration runs and an all-out 10 m sprint. Considering match demands and most reported test distances in the literature a 30 m linear sprint was measured [34]. For the timing of the 30 m sprint (with a section of 15 m as a measure for the acceleration [34]), light gates (Smartspeed, Vald Performance, Newstead, Australia) were used. The starting point was set 0.5 m in front of the light gates to avoid an early release that could occur inadvertently by a hand or stick movement or by bending the upper body forward at the start. The height of the light gate was set to 90 cm. The athletes started with complete ice hockey equipment including the stick without an external signal. After two attempts with a rest period of three minutes in between, the best 15 m and 30 m times were used for further analysis. For the determination of the flying 15 m (15 to 30 m segment time; this variable is based on match demands, because various sprint performances are generated from a submaximal speed), the sprint with the best 30 m final time was taken. The best attempt of each test is listed on the data sheet in the appendix. For on-ice sprints, the interclass reliability has been reported to be ICC = 0.86 in previous research [35].

### Statistical analysis

The data were analysed using SPSS 28.0 (IBM). The significance level was set to < 0.05 for all statistical tests. In descriptive statistics, ensemble mean, standard deviation (SD) and 95% confidence intervals (95% CI) were calculated. The Shapiro-Wilk test was used to test the data’s distribution for normality.

The relationships between the SJ, CMJ, CMJ 40%, relative and absolute strength and the on-ice sprint tests were evaluated using the one-tailed bivariate Pearson correlation coefficient for the entire sample and differentiated for the respective age groups (U16, U18, U21, Pro). The explained variance (r^2^) was calculated by squaring r. Additionally, 95% CI for correlation coefficients were reported. To determine significant differences in the correlation coefficients between subgroups (U16 vs. U18; U16 vs. U21; U16 vs. Pro; U18 vs U21; U18 vs. Pro; U21 vs Pro), the data were z-transformed according to the Fisher method. The difference of the two transformed values after standardization was tested for significance:

$$Z=\frac{\text{z}\prime_{1-}\text{z}\prime_{2}}{\sqrt{\frac{1}{{\text{n}}_{1}-3}+\frac{1}{{\text{n}}_{2}-3}}}$$

Further, the Benjamin-Hochberg method was applied to control the false discovery rate. Also the Benjamin-Hochberg method was used to correct for potential alpha errors [36].

Between-age-group differences were investigated using a one-way ANOVA with Bonferroni corrected post hoc tests. In addition, the effect size was determined with Hedges’ g. Post-hoc power (1-ß) was calculated via G*Power 3.1.9.6 (University Düsseldorf, Düsseldorf, Germany).

## RESULTS

The Shapiro-Wilk test result showed that all parameters were normally distributed.

The mean performance and SD for all the different tests, as well as the 95% CI, are shown in (Table 1). ICC coefficients were found for all tests (0.93–1.0).

**TABLE 1:** Descriptive statistics and intraclass correlation coefficients of performance variables for total group

Tests	Mean ± SD	95 % Cls	ICC
CMJ (cm)	38.64 ± 4.87	37.62–39.65	0.99
SJ (cm)	35.86 ± 5.13	29.04–30.40	0.99
CMJ 40% BW (cm)	27.08 ± 3.84	26.28–27.88	0.99
ITBP (N/kg)	29.718 ± 3.258	29.04–30.40	1.00
ITBP (N)	2269.5 ± 467.8	2172.0–2366.9	1.00
Sprint 15 m (sec)	2.57 ± 0.87	2.55–2.59	0.93
Sprint 30 m (sec)	4.37 ± 0.15	4.34–4.44	0.95
Flying 15 m (sec)	1.80 ± 0.08	1.78–1.81	0.95

SD = standard deviation; ICC = intraclass correlation coefficient; CMJ = counter movement jump; SJ = squat jump, CMJ 40% BW = counter movement jump 40% bodyweight; ITBP = isometric trap bar pull.

Pairwise comparison of coefficients from independent samples showed that there were no significant differences (p < 0.05) between the age groups in 88 out of 90 cases. Only the correlation coefficients for the variables maximum strength and flying 15 m, between the age groups U16 and U18 (p = 0.02), as well as U16 and Pro (p = 0.02), showed significant differences. After applying the Benjamin-Hochberg method, these values also showed no significant differences between the age groups. Therefore, the correlations for the total group were calculated in Table 2. Squaring correlation coeficients showed 36-59% explained variance (r^2^) for on-ice sprint and jump performance and 12-40% for strength and on-ice sprint performance. The Benjamin-Hochberg method was also used to adjust alpha error. Significant differences for all parameters were found for each group and in all combinations. Post hoc power was calculated with 96% (lowest effect size) to 100% (highest effect size) assuming an alpha level of 0.05 for correlation analysis.

**TABLE 2:** Correlation coefficients and 95% confidence intervals for the off- and on-ice tests

Tests	Sprint 15 m	Sprint 30 m	Flying 15 m
CMJ	-0.63 (-0.76 – -0.50)	-0.77 (-0.86 – -0.69)	-0.77 (-0.86 – -0.69)
SJ	-0.60 (-0.73 – -0.47)	-0.73 (-0.83 – -0.63)	-0.72 (-0.82 – -0.62)
CMJ 40% BW	-0.61 (-0.74 – -0.48)	-0.75 (-0.84 – -0.66)	-0.75 (-0.84 – -0.66)
ITBP (W/kg)	-0.41 (-0.58 – -0.24)	-0.57 (-0.71 – -0.43)	-0.63 (-0.76 – -0.50)
ITBP (W)	-0.34 (-0.53 – -0.16)	-0.53 (-0.68 – -0.38)	-0.63 (-0.76 – -0.50)

CMJ = counter movement jump; SJ = squat jump, CMJ 40% BW = counter movement jump 40% bodyweight; ITBP = isometric trap bar pull; * < 0.05.

Table 3 shows performance variables for age groups and professional players. ANOVA revealed that the groups (U16, U18, U21, Pro) differed (p < 0.001) from each other in all test results (CMJ [F_3,87_ = 16.5, p < 0.001, η^2^ = 0.363], SJ [F_3,87_ = 13.0, p < 0.001, η^2^ = 0.311], CMJ40&BW [F_3,87_ = 18.3, p < 0.001, η^2^ = 0.387], ITBP [F_3,87_ = 40.8, p < 0.001, η^2^ = 0.584], 15 m sprint [F_3,87_ = 5.6, p = 0.002, η^2^ = 0.161], 30 m sprint [F_3,87_ = 14.7, p < 0.001, η^2^ = 0.336], flying 15 m [F_3,87_ = 23.0, p < 0.001, η^2^ = 0.443]). Results of the post-hoc test (Bonferroni) are presented in Table 4. Post hoc power was calculated with 94% (lowest effect size) to 100% (highest effect size) assuming an alpha level of 0.05 for ANOVA analysis.

**TABLE 3:** Descriptive statistics of performance variables for age groups and professional players

Tests	Mean ± SD			

	U16 (n = 20)	U18 (n = 28)	U21 (n = 22)	Pro (n = 21)
CMJ (cm)	35.00 ± 3.54	36.82 ± 3.41	40.32 ± 5.00	42.76 ± 3.77
SJ (cm)	32.85 ± 3.73	33.75 ± 3.95	37.14 ± 5.64	40.19 ± 3.72
CMJ 40% BW (cm)	23.80 ± 2.98	26.00 ± 2.69	28.23 ± 3.52	30.43 ± 3.06
ITBP (N/kg)	26.54 ± 2.45	29.18 ± 2.54	31.20 ± 3.36	31.91 ± 1.88
ITBP (N)	1775.0 ± 300.2	2092.0 ± 271.6	2499.5 ± 368.1	2736.1 ± 286.2
Sprint 15 m (sec)	2.62 ± 0.09	2.58 ± 0.07	2.53 ± 0.09	2.54 ± 0.08
Sprint 30 m (sec)	4.50 ± 0.14	4.40 ± 0.11	4.30 ± 0.13	4.27 ± 0.11
Flying 15 m (sec)	1.87 ± 0.06	1.82 ± 0.06	1.77 ± 0.06	1.74 ± 0.05

n = sample size; SD = standard deviation; CMJ = counter movement jump; SJ = squat jump, CMJ 40% BW = counter movement jump 40% bodyweight; ITBP = isometric trap bar pull, U16 = under 16 years old; U18 = under 18 years old; U20 = under 20 years old; Pro = professional players.

**TABLE 4:** Effect sizes of differences between youth age groups and professional players

		CMJ	SJ	CMJ 40% BW	ITBP (W/kg)	ITBP (W)	Sprint 15 m	Sprint 30 m	Flying 15 m
U16/U18	g	0.525	0.233	0.781	1.056*	1.117*	0.502	0.755	0.947*
U16/U21	g	1.218*	0.888*	1.353*	1.570*	2.146*	1.003*	1.460*	1.735*
U16/Pro	g	2.122*	1.969*	2.193*	2.464*	3.281*	0.966*	1.752*	2.593*
U18/U21	g	0.837*	0.711*	0.726	0.690	1.464*	0.656	0.898*	0.831*
U18/Pro	g	1.665*	1.671*	1.552*	1.195*	2.316*	0.585	1.188*	1.525*
U21/Pro	g	0.551	0.635	0.666	0.261	0.715	0.106	0.191	0.576

g = effect size hedges; CMJ = counter movement jump; SJ = squat jump, CMJ 40% BW = counter movement jump 40% bodyweight; ITBP = isometric trap bar pull,

* = significant different (p < 0.05); U16 = under 16 years old; U18 = under 18 years old; U20 = under 20 years old; Pro = professional players.

## DISCUSSION

The objective of this study was to evaluate the age-dependent relationship between maximum strength and vertical power, with on-ice linear sprint performance, in elite youth and professional ice hockey players. Statistical analysis revealed that on-ice sprint performance correlates moderately to highly with isometric strength performance (r = |0.34|-|0.63|) and highly with off-ice jump performance (r = |0.61|-|0.77|). These correlations did not differ between age groups or performance levels. In contrast, however, it is noteworthy that most of the assessed performance parameters (i.e., on-ice, off-ice) differed between age groups and performance level with the largest observed differences between the youngest age group (U16) and the Pro group (g = 0.966–3.281), indicating a better performance in the older athletes.

The correlation coefficients for strength and “on-ice” performances in this study (r = |0.34–0.63|) are found to be approximately in the middle of the range of correlations stated in previous literature (r = |0.21|-|0.75|) [17–19]. The broader range of correlation coefficients in the mentioned studies may be due to a higher sampling error in these studies caused by the smaller sample sizes of *n* = 16–40. Consequently, a higher dispersion around the true coefficient can be expected in these studies, and it seems stringent to locate (roughly in the middle of the range of coefficients documented in the literature) the coefficients measured in this study, which should scatter less around the true coefficient [8].

Furthermore, the explained variance (12–40%) shows that, in addition to the influence of maximum strength, other variables (e.g., technique) can be expected to influence performance. Interestingly, the correlation coefficients of the strength performances are higher in the flying 15 m and 30 m sprint compared to the 15 m sprint. This may be explained by the high velocity specificity of muscular performance [37], as longer ground contact times (longer period of time available for the development of forces) with increasing skating speed [38–40]. Since unfamiliarity of athletes with isometric testing conditions can be assumed, it can be hypothesized that strength level using isometric testing might be underestimated [41–43]. Since the majority of the athletes perform strength and sport-specific training predominantly under dynamic conditions, the difference in contraction specificity might negatively impact correlation coefficients [44]. Not surprisingly, jumping performances have higher correlation coefficients with on-ice performances than strength performances [17, 18, 20]. However, jumping performance is also strongly dependent on strength performance [6, 8]. Nevertheless, the data obtained in this study strengthen Schmidtbleicher’s theory [45] that maximum strength, as a basic strength ability, positively influences the performance of high-speed strength. For this reason, the correlations also remain stable between the youth athletes and professionals and do not show any significant in-between differences.

The observed performance differences in favour of elite and older populations are basically consistent with findings in the literature for ice hockey [3, 4, 25–28] and other team sports [23, 46–48]. The higher performances in the elite and older subjects could possibly be attributed to the higher level of experience and training age. Playing ice hockey (and, therefore, sprinting and changing direction) may be an effective training stimulus for improving on-ice and off-ice performances. Therefore, the extent (years) to which the athletes have performed sport-specific training and additional strength and plyometric training could explain the higher performance level with increasing age. Nevertheless, athlete allocation also might influence the results, as speed-strength performance determines performance in ice hockey; as performance level (and consequently age) increases, elite sport selects primarily high-performance athletes. This study revealed that speed strength seems to be one main difference between elite and junior ice hockey players at a high performance level. The results therefore identify important factors for juniors to improve in the transition phase from junior to elite level.

This study had some limitations. Based on the study design, by investigating ice hockey teams with a fixed number of members, a sample size based on an a-priori sample size estimation was avoided. Still, based on the post-hoc G*Power analysis, the sample size of this study turned out to be sufficient for both statistical analyses (correlation and ANOVA) with power values between 94% and 100%). Another limitation of the study was that the investigated sample consisted of multiple field positions with potential differences in performance levels, which might have affected the investigated correlations. However, no goalkeeper was included in the sample as they are considered to receive training with alternative focus compared to the field players that were included in the study sample. Moreover, the inclusion of all field players from all positions might have increased the range of athletic performance levels and, thus, the generalizability of our study results. Differences in performances and influences on correlation were calculated/controlled based on calendrical age. It should be noted that the biological age was not recorded, which might have led to a bias in the above calculations (especially for the age group U16). Despite these limitations, the results of this study are valuable, providing important information about jump, maximum strength and linear sprint performance in the sport of ice hockey.

## CONCLUSIONS

The present study shows age-dependent and league-dependent performance differences in professional ice hockey players. Additionally, the study revealed the influence of maximum strength performed off-ice on on-ice performances. Strength and jumping performance should therefore be part of regular performance testing in ice hockey. Since performance differences in strength and speed-strength performances between youth teams and the Pros were evident, training of these variables is strongly recommended to improve in the transition phase from junior to elite level.

This manuscript is original and has not been previously published, nor is it being considered elsewhere until a decision is made as to its acceptability by the Editorial Review Board. This research was not supported by any funding source. The researchers have no financial interests.
